# Asystolic Cardiac Arrest Following Pneumoperitoneum From Vagal-Mediated Bradycardia During Laparoscopic Gynaecologic Surgery: A Case Report

**DOI:** 10.7759/cureus.88889

**Published:** 2025-07-28

**Authors:** Samir Petker, Gamal Ahmed

**Affiliations:** 1 Anaesthesia, West Herfordshire Hospitals NHS Foundation Trust, London, GBR; 2 Anaesthesia, Luton and Dunstable University Hospital NHS Foundation Trust, Luton, GBR

**Keywords:** anaesthesia-induced bradycardia, asystolic cardiac arrest, bilateral salpingo-oophorectomy, carbon dioxide pneumoperitoneum, gynaecological laparoscopy, high vagal tone response, intraoperative complications, laparoscopic surgery, peritoneal insufflation, vagal-mediated bradycardia

## Abstract

Vagal-mediated bradycardia is a rare adverse reaction to peritoneal insufflation during laparoscopic procedures. We report an occurrence of vagal-mediated bradycardia during an elective gynaecological procedure, which resulted in an asystolic cardiac arrest.

A 55-year-old female patient underwent an elective laparoscopic bilateral salpingo-oophorectomy for multiple symptomatic fibromas. She had no significant past medical history. She had one previous general anaesthetic for a hysteroscopy two years prior. This was carried out under a supraglottic airway device with no documented complications. Systematic clinical examination was also unremarkable, and preoperative routine bloods showed no significant abnormalities. Induction of anaesthesia was uncomplicated and unremarkable. On initiation of peritoneal insufflation, the patient had an instantaneous and significant sinus bradycardia that did not respond to boluses of atropine. She subsequently had an asystolic cardiac arrest. Return of spontaneous circulation occurred on deflation of her peritoneum. The multidisciplinary team (MDT) decision was to terminate the surgery. The patient remained stable post-operatively, and all cardiac investigations were normal.

Laparoscopic procedures entail manipulation of pelvic structures and abdominal nerves, notably during peritoneal carbon dioxide insufflation. Severe vagal reactions have been shown to occur, and this can lead, not uncommonly, to an asystolic cardiac arrest.

Preventative recommendations currently include limiting peritoneal pressure to 15 mmHg during insufflation, pre-medicating with vagolytic agents, and careful consideration of co-morbid risk factors. Treatment options focus on the intraoperative cardiac arrest protocol outlined by the Association of Anaesthetists of Great Britain and Ireland (AAGBI) and supportive care, including immediate termination of further gas insufflation and deflation of the abdomen. Atropine can also be used to treat bradycardia.

Anaesthetists should be aware of this life-threatening adverse reaction and understand risk factors, preventative measures and treatment options available during laparoscopic procedures.

## Introduction

Since the advent of laparoscopic procedures in 1985, when German surgeon Muhe carried out his first laparoscopic cholecystectomy [1], there has been a tide of change within the surgical field. Laparoscopy has now become the preferred approach for a wide range of both elective and emergency procedures. This is most apparent across the general surgical and gynaecological fields [2].

Laparoscopy renders better patient outcomes, notably smaller wounds and lower rates of post-operative complications. Studies have shown reduced rates of blood loss, post-operative pain, wound infections and wound dehiscence [3]. In comparison to open surgery, patients undergoing laparoscopic procedures also experience reduced heat and insensible fluid loss. Furthermore, there are significant differences in rates of deep vein thrombosis and chest infections related to prolonged inactivity as patients are encouraged to mobilise in a timely manner [4]. The culmination of these individual benefits results in an overall shorter patient recovery period, enhancing their post-operative experience and reducing length of hospital stay [5]. 

However, laparoscopic procedures are not without their associated risks. First, they pose a greater technical challenge in comparison to their open counterparts. Secondly, the length of the procedure has been shown to have increased [6]. They also have specific risks, notably certain vascular injuries, gas embolism, and arrhythmias [2]. One particularly rare complication of laparoscopy is vagal-mediated bradycardia. This was first reported in the 1970s, with only a few cases documented in the fields of gynaecology and general surgery since then [7]. Vagal-mediated bradycardia has been linked to asystolic cardiac arrest [7], and it is therefore prudent to be aware of its risk factors, treatment options and preventative measures. 

This case report highlights a low-risk laparoscopic bilateral salpingo-oophorectomy of a 55-year-old female patient, during which she suffered severe bradycardia that progressed into an asystolic cardiac arrest, following peritoneal insufflation. This report aims to highlight the importance of early recognition, rapid intervention and multidisciplinary preparedness in managing this rare but critical complication.

## Case presentation

A 55-year-old woman was scheduled for an elective laparoscopic bilateral salpingo-oophorectomy for multiple fibromas. The indication for the procedure was worsening symptoms in the form of pelvic pain and intermittent abdominal swelling. On the morning of her procedure, she was seen in the pre-operative assessment clinic, where she was noted to have unremarkable physical observations. She was reviewed by a junior anaesthetist who procured a full medical history and carried out a systematic clinical examination. The patient had a past medical history of folate deficiency, for which she was taking regular folic acid as prescribed by her general practitioner. She had a non-anaphylactic allergy to penicillin, where she developed a widespread rash on administration. Her social history included an office-based occupation, and she denied the consumption of alcohol or cigarettes. She carried out occasional physical activity, including 30 minutes of gentle swimming once weekly. She had undergone one previous general anaesthetic for a hysteroscopy two years prior. This was carried out under a supraglottic airway device with no documented complications.

**Figure 1:**
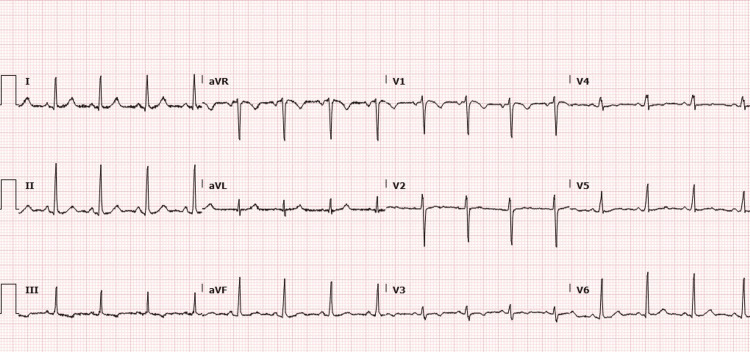
ECG from the pre-operative assessment Normal sinus rhythm at 96 beats per minute seen in the patient's preoperative assessment ECG at the clinic.

Systematic clinical examination was also unremarkable, other than a mildly elevated BMI of 28. Preoperative routine bloods showed no significant abnormalities (Table 1), and an ECG showed normal sinus rhythm (Figure 1). She consented for a general anaesthetic and was recorded as having an American Society of Anesthesiologists physical status classification of 2 (ASA-2) [8].

**Table 1:** Laboratory results of the preoperative assessment

Test	Result	Normal Range
Haemoglobin (Hb)	132 g/L	120–160 g/L
White Cell Count (WCC)	6.4 × 10⁹/L	4.0–11.0 × 10⁹/L
Platelets	258 × 10⁹/L	150–400 × 10⁹/L
Mean Corpuscular Volume (MCV)	87 fL	80–100 fL
Urea	5.1 mmol/L	2.5–7.8 mmol/L
Creatinine	68 µmol/L	45–90 µmol/L (female)
eGFR	>90 mL/min/1.73m²	>60 mL/min/1.73m²
Sodium	139 mmol/L	135–145 mmol/L
Potassium	4.2 mmol/L	3.5–5.0 mmol/L
Chloride	102 mmol/L	95–105 mmol/L
ALT (Alanine Transaminase)	24 IU/L	<35 IU/L (female)
ALP (Alkaline Phosphatase)	72 IU/L	30–130 IU/L
Bilirubin (Total)	11 µmol/L	<21 µmol/L
INR	1	~1.0
Prothrombin Time (PT)	12.6 seconds	11–13.5 seconds
ECG	Normal sinus rhythm	—
BMI	28 kg/m²	18.5–24.9 (normal), 25–29.9 (overweight)

Upon her arrival in the operating room, the patient was moved immediately to the operating table, where basic monitoring devices were attached. The observations were as follows: her blood pressure (BP) was noted to be 117/81, heart rate (HR) was 90 beats per minute (bpm), oxygen saturation (SpO2) was 97%, respiratory rate (RR) was 15, and temperature was 36.8 degrees Celsius. The HR and SpO2 were measured continuously, and the BP was set to cycle every five minutes. An 18G cannula was inserted and a litre of Lactated Ringer's was commenced.

Pre-oxygenation occurred for 3 minutes via a face mask prior to induction; 100 micrograms of fentanyl and 200 milligrams of propofol were the selected induction agents. Adequate muscle relaxation was achieved with 50 milligrams of rocuronium. Intubation was carried out two minutes later with a size 7 endotracheal tube under direct laryngoscopy that showed a grade one view. The tube was secured at 22 cm at the lips, and placement was confirmed with precordial auscultation and end-tidal carbon dioxide monitoring.

Mechanical ventilation was initiated using volume auto-flow control mode. Tidal volumes were set to 450 millilitres with a respiratory rate of 10 breaths per minute and a positive end-expiratory pressure (PEEP) of 4 mm H2O. The tidal volumes were achieved with a peak inspiratory pressure of 15 mm H2O. Anaesthesia was maintained via sevoflurane mixed with oxygen and air. Depth of anaesthesia was monitored indirectly through the maintenance of a target minimum alveolar concentration (MAC) of 1. The patient was repositioned into the lithotomy position, and the surgeons were informed that the patient was ready for the procedure to commence. All observations were noted to be within the normal range with nil complications throughout this initial anaesthetic sequence.

The surgical procedure subsequently commenced. A Veress needle technique was used to gain access to the abdominal cavity. Peritoneal insufflation subsequently began, using the pre-set pressure of 12 mmHg. Instantaneously, the HR was witnessed to rapidly drop, from 85 to 45 within a few seconds. The anaesthetist immediately administered a pre-filled syringe of 500 micrograms of atropine, followed by a saline flush. During this time, the HR continued to fall with the next reading at 12 bpm. A second dose of 500 micrograms of atropine was administered approximately 15 seconds later, by which point the monitor showed asystole.

A pulse check revealed no carotid pulses, and CPR immediately began. The cardiac arrest alarm was sounded, and the surgeons were informed to deflate the abdomen. Simultaneously, the patient was transferred from the lithotomy position to the supine position. By the time the cardiac arrest trolley was brought into the theatre and the defibrillator pads attached for the first rhythm check, approximately 30 seconds later, the patient was producing a pulse, and the monitor showed an HR of 55 in sinus rhythm. CPR was terminated, and BP was measured at 131/87. A look through the vital monitoring data at this time revealed that SpO2 was maintained at 100% throughout this period, and the end-tidal carbon dioxide (etCO2) had remained unchanged at 4.5 kPa.

A multidisciplinary team discussion took place between the surgeons, anaesthetists and intensive care team, where the decision was made to terminate the surgery given its elective nature in combination with the risk of re-insufflation of the peritoneum. The patient was extubated in the operating room, following reversal of the rocuronium, and transferred to the ward for an urgent inpatient cardiology review. Extubation was uneventful. A subsequent ECG showed normal sinus rhythm at 80 bpm, and a chest X-ray was also unremarkable, with no evidence of rib fractures or pneumothoraces. 

Cardiology carried out an inpatient echocardiogram, which once again showed no abnormalities. A diagnosis of vagal hyperstimulation secondary to peritoneal insufflation was made, and the patient was referred to the high-risk anaesthetic clinic for reassessment prior to a further attempt at the procedure.

## Discussion

This case report highlights a rare but potentially fatal complication of laparoscopic surgery: vagal-mediated bradycardia progressing to asystolic cardiac arrest following peritoneal insufflation. While the advantages of laparoscopic surgery are well documented, including reduced postoperative pain, faster recovery, and shorter hospital stays [3], its associated risks, though infrequent, must be adequately recognised and managed.

Laparoscopic procedures inherently involve physiological changes due to pneumoperitoneum creation, which is essential for operative visualisation. Carbon dioxide insufflation, typically at pressures between 10-20 mmHg [9], is known to trigger a complex interplay of autonomic nervous system responses. These include increased vagal tone, leading to bradycardia, and in severe cases, asystole. The rapid onset of bradycardia in this patient, occurring almost immediately after insufflation, underscores the direct effect of increased intra-abdominal pressure on vagal stimulation, as supported by previous literature [10].

Pathophysiology of Vagal-Mediated Bradycardia

The vagal response during laparoscopic surgery arises due to direct stimulation of the peritoneum or mesenteric structures. Increased intra-abdominal pressure and associated stretching of the peritoneum activate mechanoreceptors, which then stimulate the vagus nerve [11]. This response is exacerbated in patients with heightened vagal tone or low physiological reserve [11]. Additionally, factors such as patient positioning and depth of anaesthesia can amplify this effect. In this case, the lithotomy position may have further contributed by reducing venous return, intensifying vagal stimulation and potentiating hemodynamic instability.

Clinical Management

The timely recognition and management of bradycardia and asystole were critical to the favourable outcome in this case. Atropine administration is the first-line treatment for vagal-mediated bradycardia, acting as a muscarinic antagonist to counteract excessive parasympathetic activity. Although the initial atropine bolus was insufficient to prevent progression to asystole, rapid deflation of the abdomen and cardiopulmonary resuscitation restored circulation.

Several factors may have contributed to the failure of atropine to prevent asystole in this case. Although the AAGBI guidelines recommend a 600 mcg dose of atropine for bradycardia [12], the hospital trust supplies 500 mcg prefilled minijets, which was the dose administered at the time. Its effect may have been delayed due to the patient’s markedly reduced cardiac output, which can limit drug delivery from peripheral lines unless accompanied by a generous fluid bolus. Additionally, the timing of administration relative to the onset of bradycardia may have been too late to interrupt the escalating vagal response. These factors highlight the importance of early recognition, timely administration, and effective drug delivery when managing severe vagal-mediated bradycardia.

In patients who remain in an unstable bradycardia despite medical treatment, transcutaneous pacing should be commenced. If this too remains unsuccessful, a plan should be made for urgent transvenous pacing via a pacing wire. The multidisciplinary decision to terminate the procedure in this case was prudent, prioritising patient safety over surgical goals. Following this event, she was reviewed by a cardiologist undergoing further investigations in the community prior to rescheduling for the procedure. All investigations came back unremarkable, and the procedure was attempted for a second time six months later, this time uneventfully.

During the repeat procedure, specific precautions were taken to minimise the risk of another vagal event. Multiple 600 mcg boluses of atropine were pre-drawn and readily available, transcutaneous pacing pads were applied prior to induction, and the resuscitation trolley was brought into the operating theatre in advance. In addition, insufflation was performed more gradually with lower intra-abdominal pressure. These measures, along with increased vigilance and team preparedness, likely contributed to the uneventful outcome. This experience highlights the importance of tailored perioperative planning and readiness when re-operating on patients with a history of intraoperative vagal events.

Risk factors and prevention

Understanding patient-specific risk factors is essential for preventing similar adverse events. In this case, the patient had no significant comorbidities or prior complications from anaesthesia, suggesting that the episode was likely triggered by the mechanical effects of peritoneal insufflation rather than underlying cardiac pathology. Nonetheless, heightened vigilance is warranted in patients with predisposing conditions, such as autonomic dysfunction, obesity, or prior vagal reactions.

Preventative strategies include limiting insufflation pressures to the minimum required for adequate visualisation, ensuring gradual insufflation, and maintaining normovolaemia to counteract the hemodynamic effects of pneumoperitoneum. Prophylactic administration of anticholinergic agents like glycopyrrolate may be considered in patients with a known history of vagal-mediated responses. In patients who have had previous episodes of severe vagal-mediated responses, such as in this case, it may be prudent to carry out any future laparoscopy with transcutaneous pacing pads in situ.

Implications for practice

This case emphasises the need for heightened awareness and preparedness for rare but severe complications during laparoscopic procedures. Regular training in advanced life support and the importance of interdisciplinary communication cannot be overstated. Furthermore, patients with a history of adverse vagal responses should undergo thorough preoperative assessment and counselling regarding the risks of laparoscopic surgery.

A similar case was described by Heyba et al. (2020), where a patient experienced bradycardia and impending cardiac arrest following rapid peritoneal insufflation during laparoscopic surgery [13]. Their report supports the need for immediate desufflation and atropine administration in managing vagal-mediated events of this nature.

In resource-limited settings, implementing some of the preventive strategies described, such as advanced intraoperative monitoring, transcutaneous pacing, or immediate cardiology follow-up, may be more challenging. However, early identification of at-risk patients through clinical history and basic assessment remains essential. Where resources are limited, simple but effective steps, like having anticholinergic agents, such as pre-drawn atropine, ensuring the resuscitation trolley is immediately accessible and closely monitoring heart rate during insufflation, can still significantly impact outcomes. Conducting a preoperative team briefing to plan for potential complications can further improve preparedness, even in the absence of advanced equipment.

Future considerations

While the recurrence of such events in subsequent surgeries remains uncertain, referral to a high-risk anaesthesia clinic and preoperative cardiology evaluation were appropriate steps in this patient’s management. Future laparoscopic procedures should involve close hemodynamic monitoring and readiness to convert to an open approach if necessary. Research into alternative insufflation techniques, such as gasless laparoscopy, may further mitigate these risks in high-risk patients.

## Conclusions

This case report highlights an incident of vagal-mediated bradycardia secondary to laparoscopic peritoneal insufflation. We have a clear understanding that vagal response to rapid peritoneal stretch is a significant contributing factor. Our current management focuses on supportive management. However, we are perpetually evolving our laparoscopic techniques, and therefore should further hone peritoneal insufflation parameters. Although most patients recover without significant harm, it is evident that anaesthetists should be aware of this life-threatening adverse reaction and understand risk factors, preventative measures and prompt treatment interventions during laparoscopic procedures. This case also underscores several key learning points: the importance of early detection of bradycardia, immediate deflation of the pneumoperitoneum in the event of hemodynamic instability and the critical role of team preparedness in managing intraoperative emergencies. Reinforcing these practices can significantly improve patient safety during laparoscopic surgery.
